# Pharmacotherapy of borderline personality disorder: what has changed over two decades? A retrospective evaluation of clinical practice

**DOI:** 10.1186/s12888-019-2377-z

**Published:** 2019-12-12

**Authors:** Charles Timäus, Miriam Meiser, Borwin Bandelow, Kirsten R. Engel, Anne M. Paschke, Jens Wiltfang, Dirk Wedekind

**Affiliations:** 1 Department of Psychiatry and Psychotherapy, University Medical Centre, University of Goettingen, Von-Siebold-Straße 5, 37075 Goettingen, Germany; 2 German Center for Neurodegenerative Diseases, Goettingen, Germany; 3 Institute for Research in Biomedicine, Medical Science Department, University of Aveiro, Aveiro, Portugal

**Keywords:** Borderline personality disorder, Pharmacotherapy, Comorbidity

## Abstract

**Background:**

The purpose of this study was to assess the pharmacological treatment strategies of inpatients with borderline personality disorder between 2008 and 2012. Additionally, we compared pharmacotherapy during this period to a previous one (1996 to 2004).

**Methods:**

Charts of 87 patients with the main diagnosis of borderline personality disorder receiving inpatient treatment in the University Medical Center of Goettingen, Germany, between 2008 and 2012 were evaluated retrospectively. For each inpatient treatment, psychotropic drug therapy including admission and discharge medication was documented. We compared the prescription rates of the interval 2008–2012 with the interval 1996–2004.

**Results:**

94% of all inpatients of the interval 2008–2012 were treated with at least one psychotropic drug at time of discharge. All classes of psychotropic drugs were applied. We found high prescription rates of naltrexone (35.6%), quetiapine (19.5%), mirtazapine (18.4%), sertraline (12.6%), and escitalopram (11.5%). Compared to 1996–2004, rates of low-potency antipsychotics, tri−/tetracyclic antidepressants and mood stabilizers significantly decreased while usage of naltrexone significantly increased.

**Conclusions:**

In inpatient settings, pharmacotherapy is still highly prevalent in the management of BPD. Prescription strategies changed between 1996 and 2012. Quetiapine was preferred, older antidepressants and low-potency antipsychotics were avoided. Opioid antagonists are increasingly used and should be considered for further investigation.

## Background

A large epidemiologic study published in 2008 and a revised study in 2010 estimated the lifetime prevalence of borderline personality disorder (BPD) at 5.9% or 2.7%, respectively [1, 2]. Epidemiologic data show a high prevalence of patients with BPD in the medical care system. BPD is found in 50% of inpatients having attempted to suicide in the last two previous years and in 15–28% of all patients in psychiatric outpatient clinics or hospitals [3–5]. To date, psychotherapy is proclaimed as a first-line treatment of BPD. However, effects of established psychotherapies are still small and inflated by risk of bias and publication bias [6]. Psychotropic drugs such as antidepressants, antipsychotics and mood stabilizers are seen as adjunctive therapy at best, as there are still no convincing effects on BPD psychopathology [7, 8]. Furthermore, there is no approved drug for BPD in Europe and the United States. Clinical guidelines recommendations are inconsistent. Therefore, drug treatment of BPD with its heterogenous, often fluctuating symptoms and frequent comorbidities remains a challenge. A Cochrane systematic review by Lieb et al. in 2010 emphasized the need for more appropriate clinical studies [9]. Since that review, a follow-up review in 2017 could only identify five additionally placebo controlled randomized trials [10]. Thus, there is still a lack of evidence supporting changes to prescribing guidelines for BPD. The long-term course of BPD seems to be more benign than expected [11]. Therefore, all studies reporting long-term efficacy of treatments for BPD have to be seen in the light of spontaneous remission. Nevertheless, the disease leads to high burden, reduction in psychosocial functioning and increased mortality. Moreover, direct and indirect costs to society are enormous and thus better treatment strategies are needed [12].

In this report, an analysis of the development of prescription rates of psychotropic drugs in BPD in an university department of psychiatry was conducted and findings of this naturalistic study with regard to 2008–2012 are presented. Also, sociodemographic characteristics, documentation of diagnostic criteria and comorbidities of BPD inpatients were analyzed. Furthermore, results were compared with a previous investigation comprising data from 1996 to 2004.

## Methods

The retrospective data of a total of 87 inpatients between 2008 and 2012 were analyzed with respect to the frequency of psychotropic drug use at time of admission and at discharge on the one hand and to the kind of substance class and their mean dosages on the other. Furthermore, we compared so far unpublished findings from 1996 to 2004 with the findings from 2008 to 2012 in order to answer the question if pharmacological strategies changed and whether they are in accordance with the evidence from randomized controlled studies and the recommendations of available guidelines for treatment of BPD. Furthermore, data on sociodemographic characteristics, documentation of diagnostic criteria and comorbidities in BPD inpatients and the results were compared with the period 1996–2004.

### Patient selection

Consecutive inpatients of the interval 1996–2004 and 2008–2012 with a main diagnosis of BPD and a minimum age of 18 years were included. Diagnoses (F60.3) were based on the ICD-10 [13]. Patients were identified using the electronic database of the Department of Psychiatry and Psychotherapy of the University Medical Centre Goettingen, Germany. Inpatients without BPD as a main diagnosis were excluded. Furthermore, an inpatient treatment shorter than 1 week was an exclusion criterion.

### Data acquisition

The data of interest were derived from patient charts and collected on a protocol sheet before digitalized. Inpatient treatment periods, all comorbidities, documentation of diagnostic criteria for BPD and sociodemographic parameters were assessed. History of suicide attempts and reason of admission (multiple answers were allowed) were provided considering the cohort of the interval 2008–2012.

### Statistical analysis

Comparisons for nominal-scaled data were done using Fisher’s exact test. Significance was assumed if *p* < 0.05. A Bonferroni correction was applied for multiple comparisons. Evaluations were done using Graph Pad Prism Version 4.

## Results

### Description of the study cohorts

158 patients with BPD treated from 2008 to 2012 were screened for inclusion and exclusion criteria. A total of 87 patients was finally eligible. 71 patients were not eligible as BPD was not main diagnosis or no further data were available. 86.2% were female subjects. The mean age of time of first admission was 30.5 (± 10.7) years with a range of 19 to 61. The mean duration of hospital stay was 22.7 (± 34.6) days. With respect to the period 1996 to 2004 we found 142 patients being eligible for inclusion out of a total of 198 treated patients. 56 patients were excluded as BPD was not main diagnosis, patients were exclusively treated in an outpatient setting or no further data were available. In this cohort 79.6% were female. The mean age of time of first admission was 28 years (±8.5) with a range of 18 to 60. The mean duration of hospital stay was 23.0 (± 32.6) days.

### Psychiatric comorbidity and suicide attempts

86 inpatients treated between 2008 and 2012 provided data of comorbidities for further analysis. With respect to one patient information about comorbidities were missing. Therefore, 79.3% of the BPD inpatients treated between 2008 and 2012 suffered from at least one additional psychiatric disorder, 46.0% of two or more and 28.7% of three or more respectively. 55% of them also suffered from substance-related disorders (abuse/dependency: 47% alcohol, 24% cannabinoids, 9% long-term use of sedatives or hypnotics, 7% opioids). 40% of the cohort suffered from adjustment and somatoform disorders. 31% of all subjects showed affective disorders, followed by adjustment disorders or posttraumatic stress disorder (28%). A history of suicide attempts was frequently seen in this BPD cohort. 56% of the subjects reported one or more suicide attempts, 54% of them reported two to three attempts and 13% four to ten, respectively (Table 1).

**Table 1:** Prevalence of comorbidities in BPD of the interval 1996–2004 compared to the interval 2008–2012

	2008–2012 (*N* = 86)	1996–2004 (*N* = 142)	
	% (n)	% (n)	Fisher’s exact (*p* value)
substance related disorder	55 (47)	79 (112)	**0.0002**
alcohol	47 (40)	54 (76)	0.3399
cannabinoids	24 (21)	27 (38)	0.7563
sedatives and hypnotics	9 (8)	32 (45)	**< 0.0001**
opioids	7 (6)	10 (14)	0.6300
adjustment and somatoform disorders	40 (34)	61 (86)	**0.0026**
affective disorders	31 (27)	42 (60)	0.1221
adjustment disorders or PTSD	28 (24)	40 (56)	0.0868
anxiety and obsessive-compulsive disorder	21 (18)	20 (29)	1.0000
eating disorder	14 (12)	34 (48)	**< 0.0001**
behavioral disorder with somatic disturbances	14 (12)	22 (31)	0.1641
emotional dysregulation in childhood	10 (9)	5 (7)	0.1791
personality and behavioral disorder	6 (5)	27 (38)	**< 0.0001**

Rates in %; N number of patients with documented comorbidities. Bonferroni correction (*p* < 0.004). Significant p-values are highlighted in bold

### Documentation of diagnostic criteria according to ICD-10

The diagnostic criteria according to ICD 10 are listed in Table 2. Almost all subjects (99%) treated between 2008 and 2012 suffered from unstable mood. Chronic feelings of emptiness were seen in 94% of the subjects. Recurrent threats or acts of self-harm were seen in 93%.

**Table 2:** Prevalence of diagnostic criteria of BPD after ICD-10 of the interval 1996–2004 compared to the interval 2008–2012

	diagnostic criteria		
	2008–2012 (*N* = 87)	1996–2004 (N = 142)	
	% (n)	% (n)	Fisher’s exact (p value)
unstable and capricious (impulsive, whimsical) mood	99 (86)	98 (139)	0.6358
chronic feelings of emptiness	94 (82)	44 (63)	**< 0.0001**
recurrent threats or acts of self-harm	93 (81)	94 (134)	1.0000
disturbances in and uncertainty about self-image, aims, and internal preferences	92 (80)	74 (105)	**0.0009**
tendency to act unexpectedly and without consideration of the consequences	89 (77)	80 (114)	0.1426
anger or violence, with inability to control the resulting behavioral explosions	72 (63)	63 (90)	0.1934
liability to become involved in intense and unstable relationships, often leading to emotional crisis	69 (60)	46 (66)	**0.0010**
excessive efforts to avoid abandonment	69 (60)	18 (25)	**< 0.0001**
marked tendency to engage in quarrelsome behavior and to have conflicts with others, especially when impulsive acts are thwarted or criticized	53 (43)	28 (40)	**0.0002**
difficulty in maintaining any course of action that offers no immediate reward	52 (45)	39 (55)	0.0566

Rates in %, N number of patients. Bonferroni correction (*p* < 0.005). Significant *p*-values are highlighted in bold

### Reasons for admission

The most frequent reasons for admission of BPD inpatients treated between 2008 and 2012 were affective disorders (96%), followed by parasuicidal behavior (94%) and suicidal tendencies (85%). Anxiety disorders (72%) and self-injuries (65%) were also often found (Table 3).

**Table 3:** Reason of admission (2008–2012)

	2008–2012
	%
affective symptoms	96
parasuicidal tendencies	94
suicidality	85
anxiety disorder	72
act of self-harm	65
suicidal attempt	31
conflicts in relationships	26
intoxication	15
withdraw procedure	11
workplace problems /job loss	10
psychotherapy procedure	4
dissocial acts	3
others	3
homeless	1

Rates in %. Total number of admissions between 2008 and 2012 was 140

### Psychopharmacological treatment

A total of 58 patients were treated with medication at time of admission. During hospitalization, the percentage of psychopharmacological treated patients significantly increased from 67% up to 94% at discharge (Table 4).

**Table 4:** Rates of psychotropic drug classes in BPD (2008–2012)

	admission *N* = 58	discharge *N* = 82	Fisher’s exact (*p* value)
antipsychotics	34.5	46	0.1639
antidepressants	50.6	67.8	0.0305
hypnotics / sedatives	29.9	24.1	0.4949
mood stabilizers	10.3	9.2	1.0000
withdrawal medication	13.8	35.6	**0.0014**
Overall	67	94	**< 0.0001**

Rates in %; N number of patients receiving psychotropic drugs; reference is number of all included patients (87). Bonferroni correction (*p* < 0.008). Significant *p*-values are highlighted in bold

Considering the entire cohort of the interval 2008–2012, the mean number of medications was 1.24 at time of admission and 1.54 at time of discharge, respectively. Considering all medicated inpatients, the mean number of medications was 1.86 (58 medicated inpatients) at time of admission and 1.54 (82 medicated inpatients) at time of discharge, respectively.

At time of admission, more than half of the present cohort were treated with antidepressants (50.6%). 34.5% received antipsychotics and 29.9% hypnotics, respectively. 10.3% were treated with mood stabilizers. At discharge the extent of antidepressant users was 67.8% and of antipsychotics users 46%, respectively. The prescription rate of antipsychotics and antidepressants increased but not significantly so. Compared with the time of admission the prescription rate of hypnotics and mood stabilizers at discharge did not significantly differ (24.1 and 9.2% respectively). 13.8% of all subjects received medication for substance dependency, mainly the opioid antagonist naltrexone, at time of admission. At discharge 35.6% of all inpatients received naltrexone.

### Classes of psychotropic drugs

The classes of psychotropic drugs and their prevalence at time of admission and at discharge are displayed in Table 5.

**Table 5:** Rates of antipsychotics and antidepressants in BPD (2008–2012)

	admission N = 30	discharge N = 40	Fisher’s exact (p value)
low-potency antipsychotics	53,3	42,5	0.4693
high-potency antipsychotics	0	5	
second-generation antipsychotics	60	60	1.0000
	admission N = 44	discharge N = 59	Fisher’s exact (p value)
tri−/tetracyclic antidepressants	13.6	13.6	1.0000
SSRI	52.3	55.9	0.8417
MAO inhibitors	2.3	0	
atypical antidepressants	31.8	42.4	0.3096

Rates in %; N number of patients receiving antipsychotics (upper section) or antidepressants (lower section); reference = N; Bonferroni correction for antipsychotics (*p* < 0.016), for antidepressants (*p* < 0.0125)

### Antidepressants

At time of admission, patients were treated mainly with serotonin reuptake inhibitors (52.3%), followed by atypical antidepressants (i.e., mirtazapine) (31.8%), TCA (tricyclic antidepressants), tetracyclic antidepressants (13.6%), and MAO inhibitors (2.3%). It is noteworthy that all MAO inhibitors were discontinued during hospitalization. The prescription rate of atypical antidepressants increased (42.4%), however, this increase was not significant. Furthermore, the frequency of usage of SSRIs (serotonin reuptake inhibitors) (55.9%) was slightly higher compared to time of admission but did not significantly so. No significant change was also seen in the use of TCAs/tetracyclic antidepressants (13.6%).

### Antipsychotics

It is remarkable that no high-potency antipsychotics but low-potency antipsychotics (53.3%) and second-generation antipsychotics (60%) were found at time of admission. At discharge 5% of this subgroup received high-potency antipsychotics. 42.5% received low-potency antipsychotics. Second-generation antipsychotics were given at the same percentage.

### Active substances

With regard to lower-potency antipsychotics, the most prescribed substance was prothipendyl (8%), followed by promethazine (3.4%). Interestingly, 19.5% of all subjects received quetiapine, followed by aripiprazole (4.6%) and olanzapine (1.1%). Sertraline (12.6%), escitalopram (11.5%) and fluoxetine (9.2%) but atypical antidepressants (mirtazapine 18.4%) were also frequently used. 10.3% of the inpatients received diazepam. For details see Table 6.

**Table 6:** Rate of medicated inpatients (2008–2012) sorted by drug classes and substances of each class

Drug classes	Rate of medicated inpatients	mean daily dosage in mg
Antipsychotics		
Lower-potency antipsychotics		
Prothipendyl	8.0%	96.4
Promethazine	3.4%	82
Chlorprothixene	2.3%	138
Higher-potency antipsychotics		
Flupentixol	2.3%	5
Atypical antipsychotics		
Quetiapine	19.5%	263.6
Ariprazole	4.6%	10
Olanzapine	1.1%	7.5
Antidepressants		
Tri−/tetracyclic antidepressants		
Trimipramine	3.4%	90.5
Doxepin	3.4%	100
Amitriptyline	2.3%	51.9
SSRI		
Sertraline	12.6%	123.5
Escitalopram	11.5%	20
Fluoxetine	9.2%	58.6
Atypical antidepressants		
Mirtazapine	18.4%	22.5
Venlafaxine	10.3%	189.5
Hypnotics / Sedatives		
Benzodiazepines		
Diazepam	10.3%	35.1
Lorazepam	9.2%	2.6
Lormetazepam	2.3%	1
Mood Stabilizer		
Valproic acid	3.4%	700
Carbamazepine	3.4%	675
Lamotrigine	2.3%	137.5
Withdrawal Medication		
Naltrexone	35.6%	74.5

Up to the three most frequent substances (rate in %) and their mean daily dosages (in mg) were given

### Drug dosages

The ranges of prescribed dosages were very wide because of dosage fluctuations (mainly due to dosing newly added substances or due to withdrawal procedures). The mean daily dosage of sertraline was 123.5 mg. The mean dosage of escitalopram was 20 mg. For details see Table 6.

### Comparison of the periods 1996–2004 versus 2008–2012

#### Sociodemographic changes

As shown in Table 7 there were fewer subjects married compared to 1996–2004. Furthermore, a higher percentage of patients did not live in a partnership and reported more frequent partner changes. Interestingly, more subjects attended secondary school but fewer subjects qualified for university entrance in Germany (high school degree). These differences were not significant though. Fewer subjects reported relatives of first-degree suffering from psychiatric diagnosis. Furthermore, we found fewer subjects reporting relatives with a possible BPD diagnosis. Considering the current occupations of the inpatients we found a high prevalence of unemployment in both cohorts. Compared to the cohort of the previous treatment period the number of inpatients of the period 2008–2012 being unemployed were fewer. But this difference and the documented increase of patients attending a vocational training were not statistically significant.

**Table 7:** Comparison of sociodemographic characteristics (1996–2004 versus 2008–2012)

	Sociodemographic characteristics		
	2008–2012	1996–2004	
	N = 87	N = 139	Fisher’s exact (*p* value)
married	9.2	23	**0.0076
partnership	27.6	59.7	** < 0.0001
	N = 70	N = 131	
frequent partner changes	32.9	16.8	*0.0126
	N = 81	N = 110	
none	7.4	9.1	***0.7946
higher secondary school	30.9	27.3	***0.6294
lower secondary school	39.5	24.5	***0.0389
high school degree	18.5	34.5	***0.0150
	N = 85	N = 138	
unemployed	32.9	45.7	****0,0689
student	10.6	8.7	****0,6438
unskilled occupation	8.2	8.7	****0.9999
skilled occupation	8.2	8.0	****0.9999
vocational training	14.1	7.2	****0,1010
	N = 85	N = 92	
relatives with psychiatric diagnosis	38.8	77.2	* < 0.0001
	N = 85	N = 58	
relatives with possible BPD	42.2	86.2	* < 0.0001

Rates in %; references is N = number of patients with complete data set. Bonferroni correction for multiple comparisons *p < 0.05 (1 test); **p < 0.025 (2 tests); ***p < 0.0125 (4 tests); ****p < 0.01 (5 tests). Significant p-values are highlighted in bold

#### Comorbidities

Compared to the period 1996–2004, significantly fewer patients suffered from substance-related disorders, eating disorders and adjustment and somatoform disorders. Also, fewer subjects with other personality disorders were recorded (Table 1).

There were no significantly changes in terms of the substance classes alcohol, cannabinoids and opioids. Fewer subjects suffered from addiction to sedatives or hypnotics in 2008–2012 compared to 1996–2004. That difference was significant (Table 1).

#### Diagnostic criteria

Compared to 1996–2004, patients who were treated in 2008–2012 showed a significantly higher prevalence of chronic feelings of emptiness (94%), disturbances in and uncertainty about self-image, aims, and internal preferences (92%), liability to become involved in intense and unstable relationships, often leading to emotional crisis (69%), excessive efforts to avoid abandonment (69%), marked tendency to engage in quarrelsome behavior and to have conflicts with others, especially when impulsive acts are thwarted or criticized (53%) (Table 2).

#### Psychotropic drug use

As provided in Table 8 the overall prescription rate of psychotropic drugs increased in this study compared to 1996–2004 (94% versus 71.8%). A significant reduction of prescriptions of mood stabilizers could be shown (9.2% versus 24.6%). Furthermore, the proportion of carbamazepine decreased while valproic acid slightly increased. 2.3% of all medicated inpatient received lamotrigine whereas no inpatient was treated with lamotrigine between 1996 and 2004. Furthermore, the percentage of medication for addiction treatment increased significantly what was explained by an increasing usage of naltrexone in BPD (35.6% vs 6.3%). The prevalence of antipsychotics, antidepressants and hypnotics / sedatives did not significantly differ between both observation periods.

**Table 8:** Prevalence of psychotropic drugs at time of discharge (1996–2004 versus 2008–2012)

	Rates of psychotropic drugs^1^		
	2008–2012	1996–2004	
	N = 87	N = 142	Fisher’s exact (*p* value)
antipsychotics	46	37.3	0.2139
antidepressants	67.8	56.3	0.0953
hypnotics / sedatives	24,1	24,6	10.000
mood stabilizers	9.2	24.6	**0.0048**
*valproic acid*	*3.4*	*2.8*	
*carbamazepine*	*3.4*	*20.4*	
*lamotrigine*	*2.3*	*0*	
naltrexone	35.6	6.3	**< 0.0001**
overall	94	71.8	**< 0.0001**
	Rates of antipsychotics^2^		
	2008–2012	1996–2004	
	N = 40	N = 53	Fisher’s exact (p value)
low-potency antipsychotics	42.5	83	**< 0.0001**
high-potency antipsychotics	5	17	0.1073
second generation antipsychotics	60	43.3	0.1438
	Rates of antidepressants^3^		
	2008–2012	1996–2004	
	N = 59	N = 80	Fisher’s exact (p value)
tri−/tetracyclic antidepressants	13.6	53.8	**< 0.0001**
SSRI	55.9	56.3	0.9702
MAO inhibitors	0	11.3	
atypical antidepressants	42.4	37.5	0.6013

^1^Rates in %; N number of patients of the cohort. Bonferroni correction (*p* < 0.008). Significant *p*-values are highlighted in bold

^2^Rates in %; reference is number of patients receiving antipsychotics (N). Bonferroni correction (*p* < 0.016). Significant *p*-values are highlighted in bold

^3^Rates in %; reference is number of patients receiving antidepressants (N). Bonferroni correction (*p* < 0.0125). Significant *p*-values are highlighted in bold

Among all patients receiving antipsychotics the usage of classical lower-potency antipsychotics significantly decreased compared to the findings 1996–2004 (83% versus 42.5%) whereas the frequency of prescription of atypical antipsychotics increase in total but that did not significantly differ between both cohorts.

Considering all patients receiving antidepressants the subjects were treated less frequently with TCA and tetracyclic antidepressant. But no significant change was seen with respect to SSRIs or atypical antidepressants. MAO inhibitors were not used any more between 2008 and 2012.

## Discussion

In this study, patient characteristics, sociodemographic factors and pharmacological treatment strategies in inpatients with BPD over two intervals, 1996–2004 and 2008–2012, respectively, were compared.

### Patient characteristics and sociodemographic characteristics

One interesting finding of this study was the higher proportion of female inpatients between 2008 and 2012 (86.2%) compared to the cohort of the previous treatment period. Moreover, chronic feelings of emptiness and identity disturbances were very frequent symptoms found in both cohorts and were significantly more frequent among BPD inpatients treated between 2008 and 2012. With respect to BPD inpatients of the interval 2008–2012 the four most frequent reasons for admission were affective symptoms, parasuicidal tendencies, suicidality and anxiety disorder. Additionally, sociodemographic data gained some surprising results which have not been reported, previously. Overall social isolation appeared to have increased over time with fewer patients living in partnerships or being married. On the other hand, an increase of frequent partner changes was reported. These changes could also be found in the documentations of BPD diagnostic criteria. BPD inpatients of the interval 2008–2012 had more often unstable relationships and showed more often quarrelsome behavior compared to the inpatients of the previous cohort. Therefore, the social adaptation level seems to have worsened. Also, fewer patients had a high school degree which is in accordance to Gescher et al. [14]. In our study, the proportions of unemployment were generally high among BPD inpatients (32.9–45.7%). Gescher et al. previously reported similar data on the occupations of BPD patients treated between 2005 and 2009 (44%) confirming the low level of social functioning of BPD patients [14].

### Comorbidities

79.3% of the BPD inpatients treated between 2008 and 2012 suffered from at least one additional psychiatric disorder and almost one third from three or more comorbid psychiatric disorders. In this study, a significantly decrease of substance-related disorders was found (79% vs. 55%) and fewer patients were addicted to sedatives or hypnotics in 2008–2012 compared to 1996–2004. Additionally, fewer patients had an adjustment and somatoform disorder (61% vs. 40%), eating disorder (34% vs. 14%) and other personality disorders (26% vs. 6%) respectively. Zanarini et al. and Grant et al. reported similar comorbid conditions considering the substance-related disorders. They found 64.1 and 50.7% BPD patients with substance use disorders respectively [15, 16]. Interestingly, the proportion of comorbid eating disorders in this study were lower compared to the one reported by Zanarini et al. (53.0%) [15].

### Pharmacotherapy in BPD

We reported a significant increase of drug use in 2008–2012 compared to a previous cohort treated between 1996 and 2004. Almost every BPD inpatients received at least one psychotropic drug at time of discharge. Considering all medicated inpatients, the mean number of medications was 1.86 (58 medicated inpatients) at time of admission and 1.54 (82 medicated inpatients) at time of discharge, respectively. Our results stand in line with previous findings by Knappich et al. reporting results of a survey among psychiatrists in private practices in Germany investigating their prescription strategies with regard to BPD patients and they found that the overall rate of psychotropic drug therapy was 94% [17]. Data from the European Drug Safety Project AMSP revealed that 90% received at least one medication [18]. The high prevalence of psychopharmacotherapy is also true for other European countries (92% in the UK) [19] and the United States [20].

### Antidepressants

Compared to the later period 1996–2004, fewer subjects received TCAs or tetracyclic antidepressants and treatment with MAO inhibitors was completely abandoned, maybe due to the better tolerability of SSRIs and atypical antidepressants but also because of the toxicity of TCA when overdosed, i.e. during a suicide attempt. In our study, SSRIs were the leading antidepressants. Also, Bridler et al. published that about 70% of the BPD patients received antidepressants, predominantly SSRIs (39.2%) [18]. Actually, SSRI showed positive effects in terms of alleviating affective and maybe impulsive symptoms too [21] and sertraline could be superior to olanzapine in terms of treating depressive symptoms [22]. Stoffers and Lieb reviewed pharmacological RCTs up to August 2015 and they found neither statistically nor clinically significant efficacy for any SSRIs [23]. They also considered previous Cochrane Collaborations reviews [8, 9] and concluded that recommendations by the APA 2001 guideline with respect to SSRIs are no more valid. Instead, SSRIs should be used to treat major depressive disorders or if “another comorbid condition required” antidepressants [9].

### Antipsychotics

The prescription rate of second-generation antipsychotics among all inpatients receiving antipsychotics was higher in 2008–2012, but that was not significantly so compared to 1996–2004. The use of lower-potency antipsychotics dropped significantly over the same time period (83% 1996–2004 versus 42.5% 2008–2012). Interestingly, we saw frequent usage of the atypical antipsychotic quetiapine (19.5%), which was in accordance with the findings of the AMSP study (mean 22% 2001–2011 and 33% 2009–2011), followed by aripiprazole (4.7%). This observation is conforming to available RCTs describing promising effects in terms of second-generation antipsychotics [21, 22], i.e. of quetiapine [24, 25], in some psychopathologies seen in BPD patients.

### Mood stabilizers

Although there was kind of evidence on the use of mood stabilizers in the treatment of BPD patients [9, 23], more recent findings oppose these former recommendations [26]. Indeed, we found a low overall prescription rate of mood stabilizers in our sample. In contrast, Bridler et al. showed threefold higher prescription rates in terms of anticonvulsants (31.5%) [18]. However, the reason for that was a lower prescription rate of carbamazepine compared to the period 1996–2004, which was initially about 20%. Only 3.4% of the inpatients with BPD received carbamazepine between 2008 and 2012. One reason for that could be the rising awareness for its troublesome pharmacological interactions. Instead, lamotrigine was used in our clinic during the last observation period. It would be of great interest if the recent findings towards the effectiveness of lamotrigine will have a significant impact on the prescription behavior.

### Sedatives

The use of benzodiazepines among BPD inpatients is still high (24.1% at discharge). With exception of short-term use this class of drugs should be considered to be obsolete because of the addictive potential [27]. Furthermore, alprazolam seems to increase suicidal tendencies and aggressive behaviors [28]. However, some patients may have been discharged with a benzodiazepine prescription because these patients were already taking these drugs at admission and could not been withdrawn abruptly. The attitude of psychiatrists towards hypnotics/benzodiazepines was investigated by Knappich et al. revealing that 71.4% of them announced to prescribe benzodiazepines, most often lorazepam, in BPD outpatients [17].

### Opioid antagonists

The use of naltrexone increased compared to the interval 1996–2004. Dysregulations of the endogenous opioid system seems to be crucial for the pathogenesis of BPD [29]. According to this theory, opioid antagonist treatment can have acute and long-term effects. Acutely, naltrexone can block the rewarding effects of harmful BPD behaviours, e.g. self-injury, Chronically, naltrexone may increase number and sensitivity of μ-opioid receptors which appear to be lowered in BPD. Although there are few controlled studies with opioid antagonists for the treatment of BPD, many studies have shown the efficacy of the opioid antagonists naltrexone and nalmefene in symptoms that are often associated with BPD, including self-harming behaviors [30–32], heroin [33], amphetamine [34] and alcohol addiction [35–37], pathological gambling [38, 39] and anorexia/bulimia nervosa [40]. Furthermore, an open label study showed reduction of dissociative symptoms in BPD females [41]. This finding could not be confirmed by a small cross-over placebo controlled study, perhaps due to the low power of the study [42]. Additionally, another RCT evaluating nalmefene in patients with BPD with comorbid alcohol abuse showed a significant reduction in heavy drinking days and a reduction in a BPD symptom list and CGI-BPD [43]. Nevertheless, the efficacy of naltrexone and other opioids antagonists needs to be investigated further.

### Limitations of the study

One limitation is the retrospective study design and the lack of matched controls. Furthermore, informations about the efficacy of the psychotropic drugs were not available. It is of note that our investigation focused on an inpatient university department and mainly on acute crisis interventions. Therefore, the results presented in this study are not necessarily representative for the entire population. However, in a comparison of our acute psychiatric department with our neighbor department for psychotherapy, our workgroup showed that the extent of usage of psychotropic drugs did not significantly differ between these two departments (unpublished data).

## Conclusions

Data from the present study show important changes considering the sociodemographic characteristics and frequency of critical symptoms of BPD patients pointing to a lower social adaption level due to more pronounced disturbances in relationship-building abilities. This finding needs to be considered as it impacts the therapy adherence of BPD patients and the therapeutic working alliance. Furthermore, it was shown that psychopharmacological treatment is a “real life”- strategy in order to manage specific targeted symptoms of BPD in a German psychiatric university department. Our findings are in line with other studies showing that polypharmacy is more likely among patients suffering from BPD than other axis II disorder subjects [44]. While general, however outdated, recommendations of the APA (American Psychiatric Association) [45] suggested a syndrome-oriented use of medication, the newer NICE-Guideline of the United Kingdom in 2009 [46], which was recently confirmed by the NICE surveillance report of personality disorders [47], and the most recent Australian NHMRC guideline do not recommend psychotropic substances as a basic therapeutic strategy in BPD per se and emphasize the superiority of psychotherapy. The WFSBP guidelines described some evidence for SSRI, e.g. fluoxetine and fluvoxamine, and second-generation antipsychotics [48]. However, as there is a lack of direct comparisons of psychotherapy and medication for BPD, little is known about the relative efficacy of these two treatment modalities and guideline committees should consider that almost every patient in clinical settings is treated with at least one psychotropic drug. It is quite common that therapists rely on psychotropic drug therapy if BPD patients were admitted to acute psychiatric settings which could partly be due to a limited accessibility of psychotherapeutic interventions. Furthermore, BPD patients hold on medications and appear to cumulate medications in context of inhospitalizations as suggested by our study showing an increasing mean number of medications from admission to discharge. Therefore, we simply do not know for sure how the course of BPD is in absence of psychotropic drug treatment. Moreover, recent findings suggest a possible iatrogenic effect of psychiatric crisis services being found as the sole predictor of the number of follow-up year suicide attempts. In that study, DBT may reduce suicide risk which was partly attributed to the fewer use of psychiatric crisis services [49]. However, the causality of the reported findings, especially in terms of drug therapy, needs to be investigated in further studies. It should also be considered that the most recent data, giving evidence on short- and long-term psychotherapy, from about 40 new randomized- controlled studies were published since 2012. Therefore, it would be of great interest to investigate to what extent they have already been introduced into the current clinical practice in the meantime [50] and if newer psychotherapy protocols are able to achieve a reduction of psychotropic drug use in the short and long term, respectively.

Our comparison of two time periods showed a significant decline in terms of prescription rates of tricyclic/tetracyclic antidepressants and low-potency antipsychotics. There is a stable high usage of second-generation antidepressants (i.e. SSRIs but also atypical antidepressants like mirtazapine). Second-generation antipsychotics are of growing interest in research studies but also in clinical practice. We found a declining use of mood stabilizers, which seems reasonable considering the weak evidence on the use of mood stabilizers. Lamotrigine was used in our clinic between 2008 and 2012. In the meantime, a recent study failed to prove a benefit of lamotrigine in BPD [26]. We showed a significant increase of the prescription rates of opioid antagonists (i.e. naltrexone). Benzodiazepines were prescribed at a lower frequency, but usage is still regularly seen in this study which is not generally supported by evidence and should be restricted to crisis interventions.

## Data Availability

All data generated or analyzed during this study are included in this published article (and its supplementary information files).

## Abbreviations

BPD: borderline personality disorder
APA: American Psychiatric Association
SSRI: serotonin reuptake inhibitor
TCA: tricyclic antidepressants
WFSBP: World Federation of Societies of Biological Psychiatry
